# Cordycepin kills *Mycobacterium tuberculosis* through hijacking the bacterial adenosine kinase

**DOI:** 10.1371/journal.pone.0218449

**Published:** 2019-06-14

**Authors:** Feng Huang, Weihui Li, Hui Xu, Huafeng Qin, Zheng-Guo He

**Affiliations:** National Key Laboratory of Agricultural Microbiology, College of Life Science and Technology, Huazhong Agricultural University, Wuhan, China; The University of Georgia, UNITED STATES

## Abstract

Cordycepin is an efficient component of *Cordyceps spp*, a traditional Chinese medicine widely used for healthcare in China, and has been recently acted as a strong anticancer agent for clinic. However, whether and how it may play a role in combating tuberculosis, caused by *Mycobacterium tuberculosis*, remains unknown. Here we report that cordycepin can kill *Mycobacterium* by hijacking the bacterial adenosine kinase (AdoK), a purine salvage enzyme responsible for the phosphorylation of adenosine (Ado) to adenosine monophosphate (AMP). We show that cordycepin is a poor AdoK substrate but it competitively inhibits the catalytic activity of AdoK for adenosine phosphorylation. Cordycepin does not affect the activity of the human adenosine kinase (hAdoK), whereas hAdoK phosphorylates cordycepin to produce a new monophosphate derivative. Co-use of cordycepin and deoxycoformycin, an inhibitor of adenosine deaminase (ADD), more efficiently kills *M*. *bovis* and *M*. *tuberculosis*. The *add*-deleted mycobacterium is more sensitive to cordycepin. This study characterized cordycepin as a new mycobactericidal compound and also uncovered a potential anti-mycobacterial mechanism.

## Introduction

Tuberculosis is caused by *Mycobacterium tuberculosis* (*Mtb*) and still globally threatens human health. In 2017, there were an estimated 10.0 million people fell ill with tuberculosis (TB), of which 457 560 had multidrug-resistant TB (MDR-TB) [1]. With the emergence of multidrug-resistant (MDR) *M*. *tuberculosis* strains, new drugs are urgently needed for control of TB.

**Fig 1:**
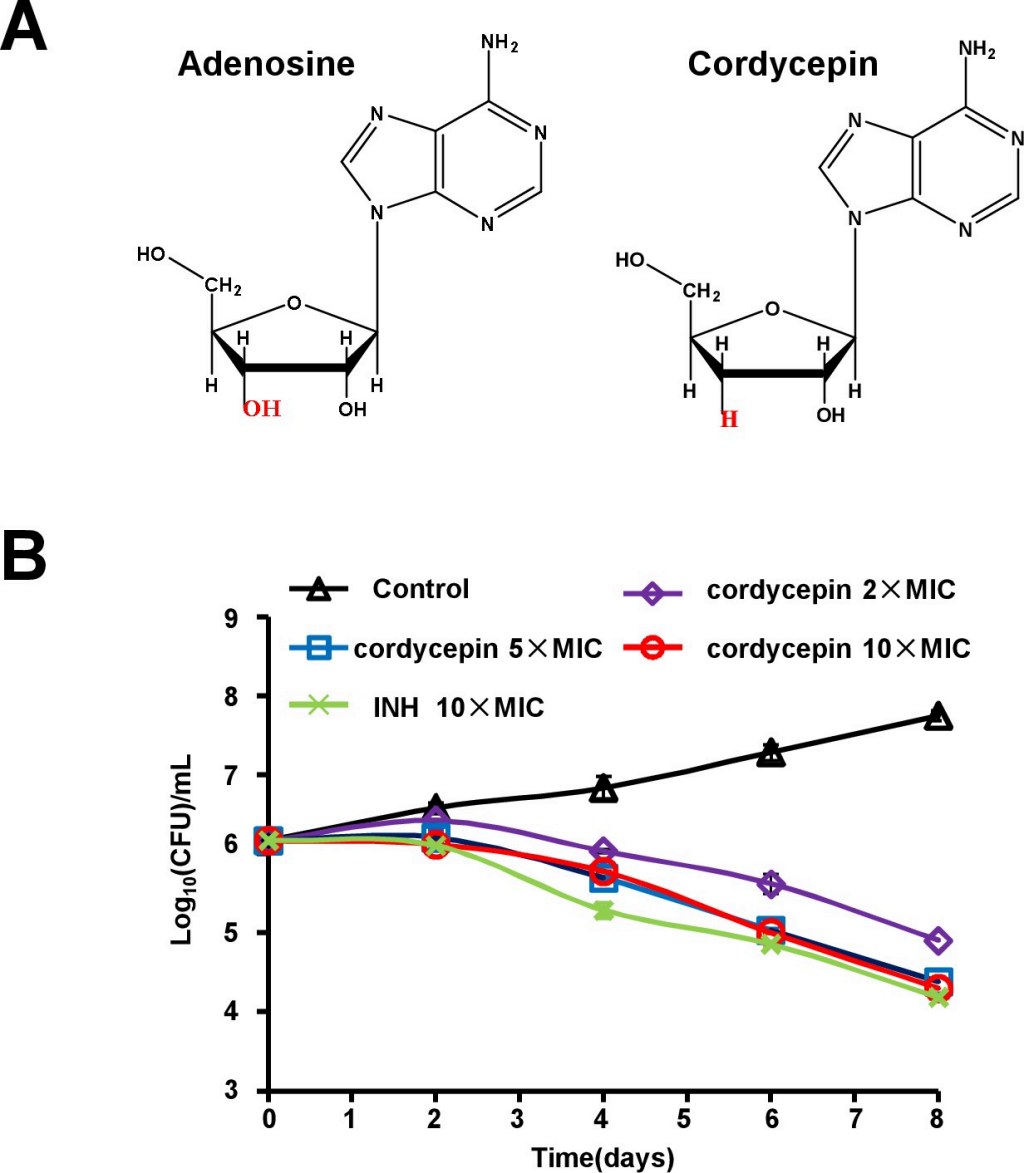
Cordycepin are active against *Mycobacterium bovis* BCG in vitro. (A) Structures of adenosine (left panel) and cordycepin (right panel). (B) Kill kinetics of cordycepin for *M*. *bovis* BCG over a period of 8 days.

Cordycepin (3′-deoxyadenosine, 3'-dAdo) is a nucleoside and structurally similar to adenosine, but it lacks a 3′-hydroxyl group (Fig 1A). Cordycepin was first isolated from the cultures of caterpillar fungus *Cordyceps militaris*, a well known Traditional Chinese Medicine widely used for healthcare in China [2]. This compound has been reported to have immunological regulation, antifungal, anti-virus, anti-leukemia, anti-inflammatory and antimetastatic effects [3–10]. In the last few years, cordycepin has been reported to have anti-tumor activity in a broad spectrum of cancer types such as Mouse Melanoma and Lung Carcinoma Cells [5], human lung cancer [11], and human brain cancer [12]. Strikingly, cordycepin can inhibit the growth of several bacterial species including *Mycobacterium avium* and *Mycobacterium bovis* [13]. However, whether and how it has a killing activity on *M*. *tuberculosis* remains to be explored.

Cordycepin is a structural homolog of adenosine and can be converted into active metabolites by adenosine kinase (AdoK), adenylate kinase and pyruvate kinase in mammalian cells [14, 15]. AdoK catalyzes phosphorylation of adenosine to adenosine monophosphate (AMP) using adenosine triphosphate (ATP) as a phosphate donor and releasing adenosine diphosphate (ADP), which is an important step in the purine salvage pathway [16]. Interestingly, AdoK is rarely found in bacteria, with the single exception of *Mycobacterium spp* [17, 18]. The activity of AdoK, enoded by *Rv2202c* (*MtbAdoK*), has been confirmed in *M*. *tuberculosis* [19]. MtbAdoK shares low structural similarity with the well-characterized human AdoKs and behaves very differently [20, 21]. MtbAdoK has been considered as a promising target for drug development [22, 23].

In the present study, we report that cordycepin can kill *Mycobacterium* by hijacking the bacterial AdoK, which suggests a potential anti-mycobacterial mechanism.

## Materials and methods

### Strains, plasmids, enzymes and reagents

*Escherichia coli (E*. *coli)* BL21 (λ DE3) and pET28a were purchased from Novagen (Darmstadt, Germany) and used to express proteins. Restriction enzymes, T4 ligase, modification enzymes, DNA polymerase, dNTPs were obtained from TaKaRa Biotech (Shiga, Japan). PCR primers were synthesized by Invitrogen (Carlsbad, USA). Ni-NTA (Ni^2+^-nitrilotriacetate) agarose was purchased from Qiagen (Hilden, Germany). 7H9 and 7H10 broths were purchased from Becton, Dickinson Company (New Jersey, USA). Cordycepin (3’-deoxyadenosine, from Cordyceps militaris, C2689) was purchased from Tokyo chemical industry CO., LTD. (Tokyo, Japan).

### Cloning, expression and purification of recombinant proteins

*adoK* (*Rv2202c*) from *M*. *tuberculosis* H37Rv genome and *hAdoK* from Homo sapiens genome were amplified by PCR using specific primers (5′-TGTAGAATTCATGTGACGATCGCGGTAACCGG-3′, and 5′- TATATATCTAGACTAGGCCAGCACGGCGACGA-3′for *adoK*,5′-ATGCGGCCGCAATGACGTCAGTCAGAGAAAA-3′ and 5′-ATGCGCTCTAGATCAGTGGAAGTCTGGCTTCT-3′ for *hAdoK*), and the mutant genes *adoK*-S115L and *adoK*-V33A were amplified by PCR using cordycepin resistant strains genome as templates. The amplified DNA fragments were cloned into the modified pET28a expression vectors to produce recombinant plasmids (S1 Table). *E*. *coli* BL21 cells were used to express the recombinant proteins. The recombinant *E*. *coli* BL21 cells were grown in 1 L Luria broth (LB) medium up to OD_600_ of 0.6. Protein expression was induced by the addition of 1 mM isopropyl β-D-1-thiogalactopyranoside (IPTG) at 16°C for 18 h. The harvested cells were resuspended and sonicated in binding buffer (20 mM Tris-HCl, pH 8.0, 500 mM NaCl, 5 mM imidazole). The lysate was centrifuged at 10000 g for 30 min, and the supernatant was loaded onto the affinity column (Ni-NTA agarose affinity matrix). The column-bound protein was washed with a wash buffer (20 mM Tris-HCl, pH 8.0, 500 mM NaCl, 50 mM imidazole). The elution was dialyzed for 2 h and stored in buffer (20 mM Tris-HCl, pH 8.0, 100 mM NaCl, 5 mM MgCl_2_, 10% glycerol) at -80°C. Protein concentration was detected using the Bradford method.

### Minimum inhibitory concentrations assays

MICs was determined using tube-broth dilution methods as previously described with several modifications [24]. For this assay, mycobacterial strains were grown in 7H9 broth without Tween-80. Other bacteria were grown in LB media. Mid-log phase culture was diluted to 5×10^6^ CFU·ml^-1^, and 0.1 ml of the dilution was used to inoculate 2.5 ml culture media containing various concentrations (0–1.2 mM) of cordycepin. Positive control (isoniazid, 0.36 μM) and growth control (no compound) were included. Each concentration has three repetitions. Tubes were incubated while shaking at 37°C for 2 weeks for slow-growing mycobacterial strains and for 1–2 days for the other bacteria. The experiments were repeated two times.

### Kill kinetic assay

Kill kinetics of cordycepin for *M*. *bovis* BCG were performed as previously described with some modifications [25]. *M*. *bovis* BCG was cultured in 7H9 broth to mid-log phase (OD600≈1.0). Bacteria were diluted to ~1.5×10^6^ CFU ml^-1^. Cordycepin was added at 0 (control), 0.12, 0.3 or 0.6 mM, isoniazid at 3.6 μM. At indicated time points, samples were taken and CFU counts were performed.

### Screening and characterization of cordycepin-resistant mutants

To select resistant mutants, 7H10 agar plates containing 5× and 10×MIC of cordycepin were prepared. Log phase *Mycobacterium bovis* (*M*. *bovis*) BCG cultures (7 ml culture, OD_600_≈1.0) were treated under the UV irradiation using the 15 W ultraviolet lamp (TUV15W/G15T8, PHILIPS, Poland) for 2.5 minutes and cultured in Middlebrook 7H9 media for 24 hours. Then, the cultures were spread on those cordycepin containing plates and incubated at 37°C for 4 weeks. Colonies were recovered and propagated in 7H9 broth containing corresponding level of the cordycepin. Genomic DNA was isolated using with a DNeasy Blood & Tissue Kit (Qiagen, Hilden, Germany) according to the manufacturer’s protocol. Whole-genome sequencing was performed using Illumina technology with sequencing libraries prepared following the protocol of SureSelectXT Target Enrichment System Paired-End Sequencing Library (Agilent Technologies, Inc. Santa Clara, USA) at Shanghai Biotechnology Corporation. Sequencing reads were aligned to the *M*. *bovis* BCG Pasteur 1173P2 reference genome with the Burrows-Wheeler Aligner (BWA, v0.7.10) with default [26]. The SNPs (*adoK*-S115L and *adoK*-V33A) identified by whole-genome sequencing were validated by Sanger sequencing.

### Construction of the *adoK* and *add* deletion mutant of *M*. *bovis* BCG

Knockout of the *adoK* and *add* gene in *M*. *bovis* BCG was performed as described previously with some modifications [27]. A pMind derived suicide plasmid carrying a hygromycin (*hyg*) resistance gene was constructed and a reporter gene *lacZ* was inserted as a selection marker. The primers for genes knock-out were shown as follows: 5′-GCGATTAATTAAGTCGATCGATTTCGTCGA-3′ and 5′- ATATACTAGTCAC AAAATCTCCGTCCTTCG-3′ for *adoK* upstream 1000 bp fragment; 5′- ATAGAAG CTTATGCGATTCCGCGTCTGCT-3′ and 5′-ATATGCTAGCAGATCGTCGGTACC T CGA-3′ for *adoK* downstream 946 bp fragment. 5′- GCGCTTAATTAAAAATC ACGCTGCCATTGGTG-3′ and 5′- ATAACTAGTCACCAGACGATCCGATCGAC GAT-3′ for *add* upstream 1000 bp fragment, 5′- GCGCAAGCTTTCAGCAAGTT CTCTGGTAT-3′ and 5′-ATATGCTAGCGAAAGGTGGAGCGCCCGTA-3′ for *add* downstream 1000 bp fragment. Genomic DNA from allelic exchange mutants in which the *adoK* or *add* gene had been deleted was identified by PCR analysis using primers of *adoK* or *add* and the *hyg* gene.

### Cordycepin sensitivity assays

The recombinant strains were grown in Middlebrook 7H9 media (supplemented with 10% OADC enrichment, 0.05% Tween-80, and 0.2% glycerol) containing 30 μg/ml Kan for a week. Cells were cultured to an OD_600_ between 1.5 and 2.0, and each culture was diluted in 100 ml fresh 7H9 broth to an OD_600_ of approximately 0.15 with or without the indicated concentration of cordycepin. The cultures were then grown at 37°C with shaking at 160 rpm. Aliquots were taken at the indicated times and plated on 7H10 medium (supplemented with 10% OADC enrichment and 0.2% glycerol) to determine colony-forming units [28]. The experiments were repeated three times.

### Adenosine kinase activity assays

The assays for adenosine kinase activity of AdoK were performed as previously described with some modifications [19]. Ten microliter reaction mixtures contain 50 mM Tris–HCl (pH 8.0), 10 mM KCl, 10 mM MgCl_2_, 12.5 μCi/ml (final concentration) [γ-^32^P] ATP, 1.5 mM ado or various concentrations of cordycepin (0.75 mM–3 mM). The activity of human Ado kinase was determined as described above with the following changes about buffer component: 50 mM Tris–HCl (pH 7.5), 40 mM KCl, 1 mM MgCl_2_, 12.5 μCi/ml (final concentration) [γ-^32^P] ATP, 1.5 mM ado or various concentrations of cordycepin (0.75 mM–3 mM). The reaction mixtures were co-incubated for 30 min at 37°C and then stopped by the addition of 50 mM EDTA. Products were separated by thin-layer chromatography (TLC) on a PEI Cellulose F -coated strip (Merck) in 1 M formic acid and 0.5 M Lithium chloride at room temperature for 40 min [29]. The TLC plate was exposed to a storage phosphor screen for 5 h. Image was obtained using FLA-5100 Scanner, and the remaining [γ-^32^P] ATP was quantified by Multi Gauge software. The experiments were repeated three times.

### HPLC and LC-MS analysis

Ado and cordycepin were used as substrates for adenosine kinase activity assays. The reaction products were detected by HPLC as described earlier with some modifications [30, 31]. The reaction buffer was the same as in the adenosine kinase activity assays with addition of 5 mM ATP and 0.5 mM Ado or cordycepin. Reactions proceeded at 37°C for 1 h and terminated by the addition of an equal volume of 1 M perchloric acid. Then, the samples were neutralized to pH 6.0 with 5 M K_2_CO_3._ The mixtures were centrifuged to remove the precipitated KClO_4_. The supernatant samples (10 μl) were injected into C-18 columns (Hypersil ODS2, 250×4.6 mm, 5μm) separated by reverse-phase HPLC (LC20AT). Buffer A (100 mM Na_2_HPO_4_, 100 mM KH_2_PO4, 5 mM Tetrabutylammonium bromide, pH6.0) and buffer B (methanol) were used at a 95:5 gradient at a flow rate of 1 ml/min. Nucleotides were detected at 259 nm wavelength. The new product synthesized by human adenosine kinase was analyzed using an Agilent 6540 Ultra High Definition (UHD) Accurate-Mass quadrupole time of flight (Q-TOF) LC-MS system. The samples (1 μl) were injected into a ZORBAX Eclipse Plus C18 (2.1×100 mm, 3.5 μm) analytical column(Agilent Technologies) operated at 30°C using 5% CH_3_OH and 95% H_2_O as mobile phases A and B, respectively, (5%-35% A in 11 min) at a flow rate of 0.3 ml/min. All samples were injected in duplicate. Experimental parameters were set as follows: fragmentor, 140V; Vcap, 4000V and gas temperature, 350°C. The scanning range of the Q-TOF was m/z 100 to 1000 [32, 33].

### Cytotoxicity assay

Lactate dehydrogenease (LDH) cytotoxicity of cordycepin or deoxyformycin was determined as previously described with some modifications [34]. Bone marrow-derived macrophages (BMDMs) were seeded at 5×10^4^ per well in 96-wells plate and incubated overnight in an incubator at 37°C, 5% CO_2_. And on the next day, cells were treated by cordycepin at the concentration of 0 (control), 0.04, 0.08, 0.32, 0.4, 0.8 and 1.6 mM or 0.4 mM deoxyformycin for 18 h. LDH activity in the media was determined using Pierce LDH Cytotoxicity Assay Kit (Thermo Scientific, USA) according to the manufacturer’s protocols.

### Intracellular survival assay

Bone marrow-derived macrophages (BMDMs) were seeded at 5×10^5^ per well in 24-wells plate and infected with *M*. *bovis* BCG at a multiplicity of infection (MOI) = 1 in DMEM/F12 medium plus 10% fetal bovine serum (FBS) as previously described with some modifications [35]. The infection was carried for 4 h, at which time the monolayer was washed three times with PBS to remove extracellular bacteria and fresh medium with inhibitor was added. After 18 h, cells were lysed with 0.05% (w/v) SDS for 10 min at room temperature and serial dilutions were plated on Middlebrook 7H10 solid medium with 10% OADC and incubated at 37°C. CFUs were enumerated after 14 days of incubation.

## Results

### Cordycepin has antibacterial activities to *M*. *bovis* and *M*. *tuberculosis*

To detect potential anti-mycobacterial activity of cordycepin, we determined and compared the minimum inhibitory concentration (MIC) of cordycepin on several mycobacterial and non-mycobacterial strains. Strikingly, cordycepin can effectively prevent the growth of *M*. *bovis* BCG, *M*. *tuberculosis* H37Ra, and *M*. *marinum* M. The MIC value for them is determined as 0.06 mM, 0.1 mM and 0.18 mM, respectively (Table 1). By contrast, no antibacterial activity was observed for the fast-growing *M*. *smegmatis* and non-mycobacterial species, including *Escherichia coli*, *Staphylococcus aureus*, and *Bacillus thuringiensis*, even the drug concentration was raised up to 12×MIC of *M*. *tuberculosis* H37Ra.

**Table 1:** MIC determination of cordycepin against selected microorganisms.

Organism and genotype	Cordycepin MIC(mM)
*M*. *bovis BCG* Pasteur 1173P2	0.06
*M*. *tuberculosis* H37Ra	0.1
*M*. *marinum* M	0.18
Cordycepin -resistant *M*. *bovis* BCG (CR01)	>1.2
Cordycepin -resistant *M*. *bovis* BCG (CR02)	>1.2
*M*. *smegmatis*	>1.2
*Escherichia coli*	>1.2
*Staphylococcus aureus*	>1.2
*Bacillus thuringiensis*	>1.2

MIC, minimal inhibitory concentration. The MIC was determined by tube-broth dilution methods.

To test the effect of cordycepin on bacterial viability, we assessed the killkinetics of cordycepin at concentrations of 2×, 5× and 10× the MIC with the BCG strain. The 5× MIC of cordycepin killed ~2 log_10_ colony forming units (CFUs) by day 8 (Fig 1B).

These results suggest that cordycepin has a specific bactericidal activity to the slow-growing mycobacterial species such as *M*. *bovis* and *M*. *tuberculosis*.

### AdoK gene mutations attribute mycobacterial resistance to cordycepin

Utilizing *M*. *bovis* BCG as an experimental strain, we further screened and identified the gene involved in the mycobacterial sensitivity to cordycepin. After UV irradiation, *M*. *bovis* BCG colonies resistant to cordycepin were selected by growing the BCG strain on 7H10 agar plates containing 10×MIC of cordycepin. We finally obtained two colonies, designated as CR01 and CR02, which can propagate when inoculated into 7H9 media containing 20×MIC of cordycepin (Table 1), suggesting that the two colonies would contain gene mutations responsible for cordycepin resistance in the mycobacterium.

We then sequenced the genomes of CR01and CR02 as well as the parental *M*. *bovis* BCG strain using Illumina sequencing technology (S2 Table). Point mutations that conferred cordycepin resistance were thereafter identified by comparing the genome sequences of the susceptible wildtype *M*. *bovis* BCG strain with two resistant mutants. Interestingly, compared to the parental strain, the only affected gene commonly in both CR01 and CR02 mutants encodes an adenosine kinase, that is AdoK which catalyzes the phosphorylation of adenosine to AMP. The point mutation was identified as S115L in CR01 and V33A in CR02 (Table 2). This SNP could be further validated by Sanger sequencing.

**Table 2:** Single nucleotide polymorphisms identified in the *Mycobacterium bovis* BCG isolates.

Sample Name	SNP description	Locus tag	Gene name	SNP class	Codon_Change	AA change	Gene length
**CR01**	**G2446328A**	**BCG_2218c/Rv2202c**	***adok***	Missense	**tCg/tTg**	**S115L**	**0.975kb**
CR01	C3234672G	BCG_2962c/Rv2940c	*mas*	Missense	cGc/cCc	R678P	6.336kb
CR01	G3427684A	BCG_3130c/Rv3105c	*prfB*	Missense	aCc/aTc	T236I	1.137kb
CR02	C2263939G	BCG_2052c/Rv2033c	*-*	Missense	Gtg/Ctg	V266L	0.843kb
**CR02**	**A2446574G**	**BCG_2218c/Rv2202c**	***adok***	**Missense**	**gTg/gCg**	**V33A**	**0.975kb**

**Fig 2:**
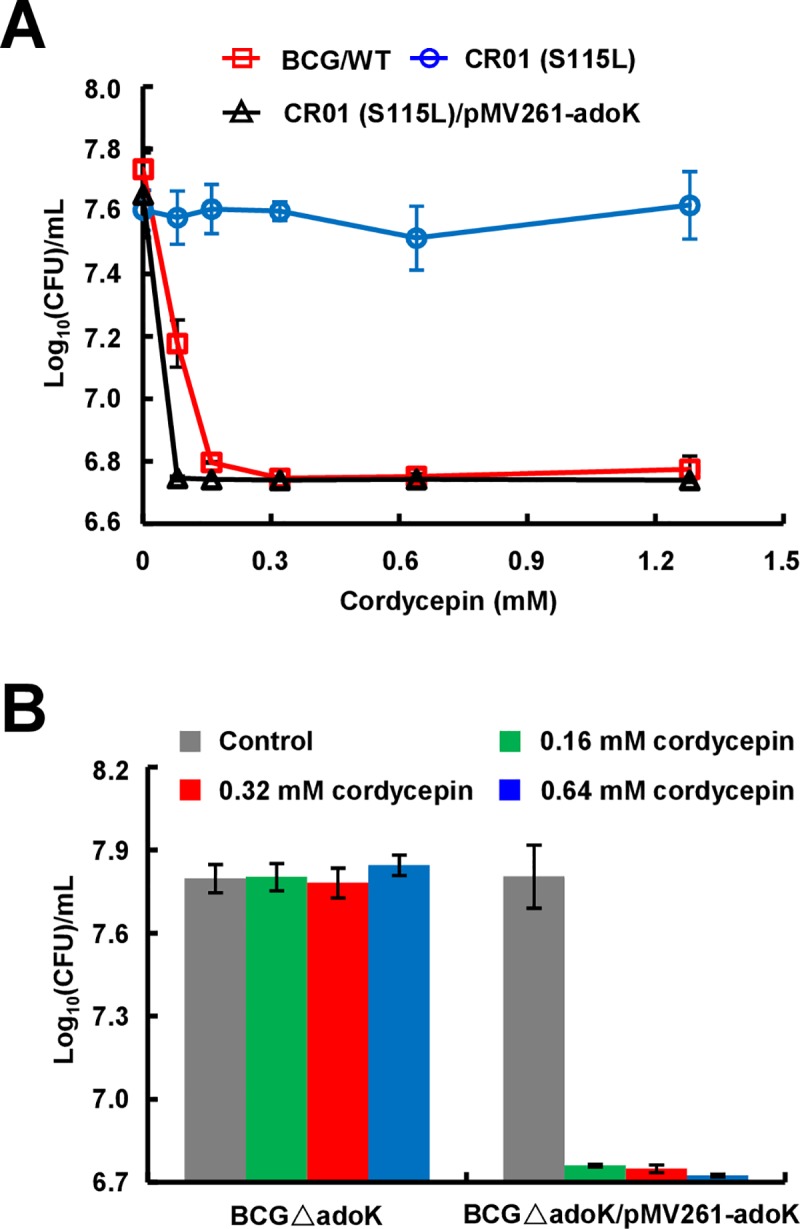
AdoK is responsible for mycobacterial sensitivity to cordycepin. (A) Mutant strain CR01-S115L is resistant to cordycepin. Mycobacterial strains were grown in 7H9 medium containing 0, 0.08, 0.16, 0.32, 0.64, or 1.28 mM cordycepin at 37°C for 9 days. Their CFUs were then determined and indicated in the figure. Both wildtype BCG/WT and the complemented strain CR01-S115L/pMV261-*adoK* are sensitive to cordycepin. (B) Assays for the sensitivities of *adoK*-deleted and its complemented strains to cordycepin. The mycobacterial strains were grown in 7H9 medium containing 0 (control), 0.16, 0.32, and 0.64 mM cordycepin at 37°C for 9 days, and CFUs were measured. All error bars in the figures represent the standard deviations (SD) of the data derived from three biological replicates.

Further complementation experiments confirmed this observation. As shown in Fig 2A and S1 Fig, when two mutant strains CR01 and CR02 were transformed with a vector expressing wildtype *adoK* gene, the recombinant strains could re-obtain a sensitivity to cordycepin, which is very similar to the phenotype of wild-type *M*. *bovis* BCG. To further validate the conclusion that the gene mutation of *adoK* is responsible for mycobacterial resistance to cordycepin, we constructed *adoK*-deleted *M*. *bovis* BCG (S2 Fig) and various complemented strains. As shown in Fig 2B, *adoK*-deleted BCG strain grew well even in 10×MIC concentration (0.64 mM) of cordycepin, whereas the *adoK*-complemented strain re-obtained sensitivity to 0.16 mM cordycepin. Strikingly, either *adoK*-S115L or *adoK*-V33A mutant gene did not affect the resistant phenotype of *adoK*-deleted *M*. *bovis* BCG (S2B Fig).

These results suggest that the *adoK* gene mutation led to the mycobacterial resistance to cordycepin.

### Cordycepin inhibits the adenosine kinase activity of AdoK

**Fig 3:**
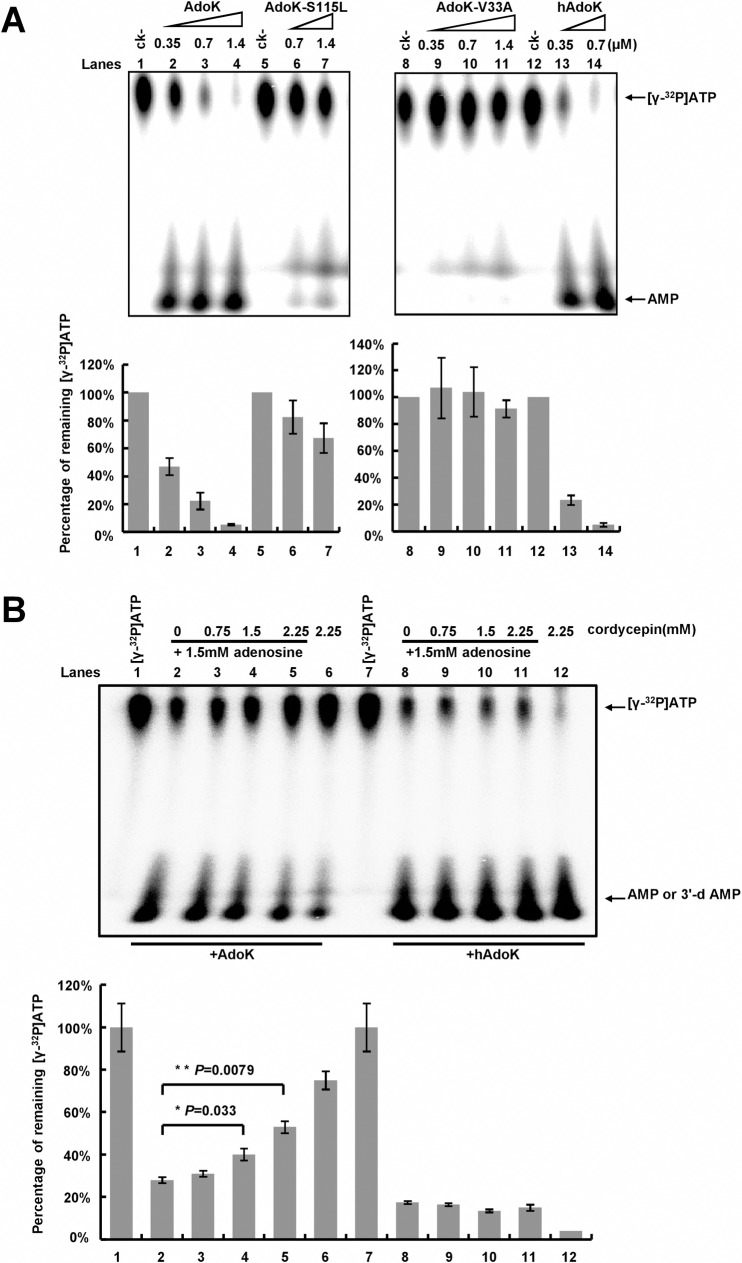
Thin-layer chromatography assays for the adenosine kinase activity and the inhibitory effect of cordycepin. (A) Assays for the kinase activities of wildtype and mutant AdoK proteins. Radioactive-labeled ATP and AMP are indicated by arrows on the right of the figure. The protein concentrations are indicated on top of the panels. Quantification assays for the percentage of remaining radioactive ATP in the reactions (upper panels) were performed and correspondingly shown as the lower panels. (B) Competitive TLC assays for the inhibitory effect of cordycepin on the activity of AdoK. An increasing concentration of cordycepin gradually inhibited the activity of AdoK, but did not for hAdoK, and the remaining ATP correspondingly increased. Relative percentages of remaining [γ-^32^P] ATP in the reaction mixtures were quantified, and the mean values of three independent experiments along with error bars (SD) are shown. The *P*-values were calculated by unpaired two-tailed Student’s *t*-test using GraphPad Prism 5. The *P*-values are indicated on top of the columns.

Next, we confirmed that both AdoK-S115L and AdoK-V33A mutant proteins lost most of their kinase activities. Using a Thin-layer chromatography assay and [γ^32^P]labeled-ATP, we observed that the AMP products gradually increased with addition of increasing amounts of AdoK proteins into the reactions, meanwhile, the radioactive ATP substrates correspondingly decreased, indicating that AdoK had adenosine kinase activity (Fig 3A, lanes 1–4). Meanwhile, we could also detect a good adenosine kinase activity for human AdoK (Fig 3A, lanes 12–14). By contrast, both AdoK-S115L and AdoK-V33A mutant proteins exhibited little activity under similar conditions (Fig 3A, lanes 5–11).

We further examined if cordycepin can act as a substrate for AdoK, or if cordycepin inhibits its adenosine kinase activity. As shown in the Fig 3B, when using cordycepin to replace adenosine as a reaction substrate, a very weak catalytic activity for AdoK was observed. By contrast, cordycepin can competitively inhibit the activity of AdoK (Fig 3B), implying that cordycepin inhibits the mycobacterial growth by interfering with the kinase activity of AdoK.

### Cordycepin is a good substrate of human AdoK kinase

**Fig 4:**
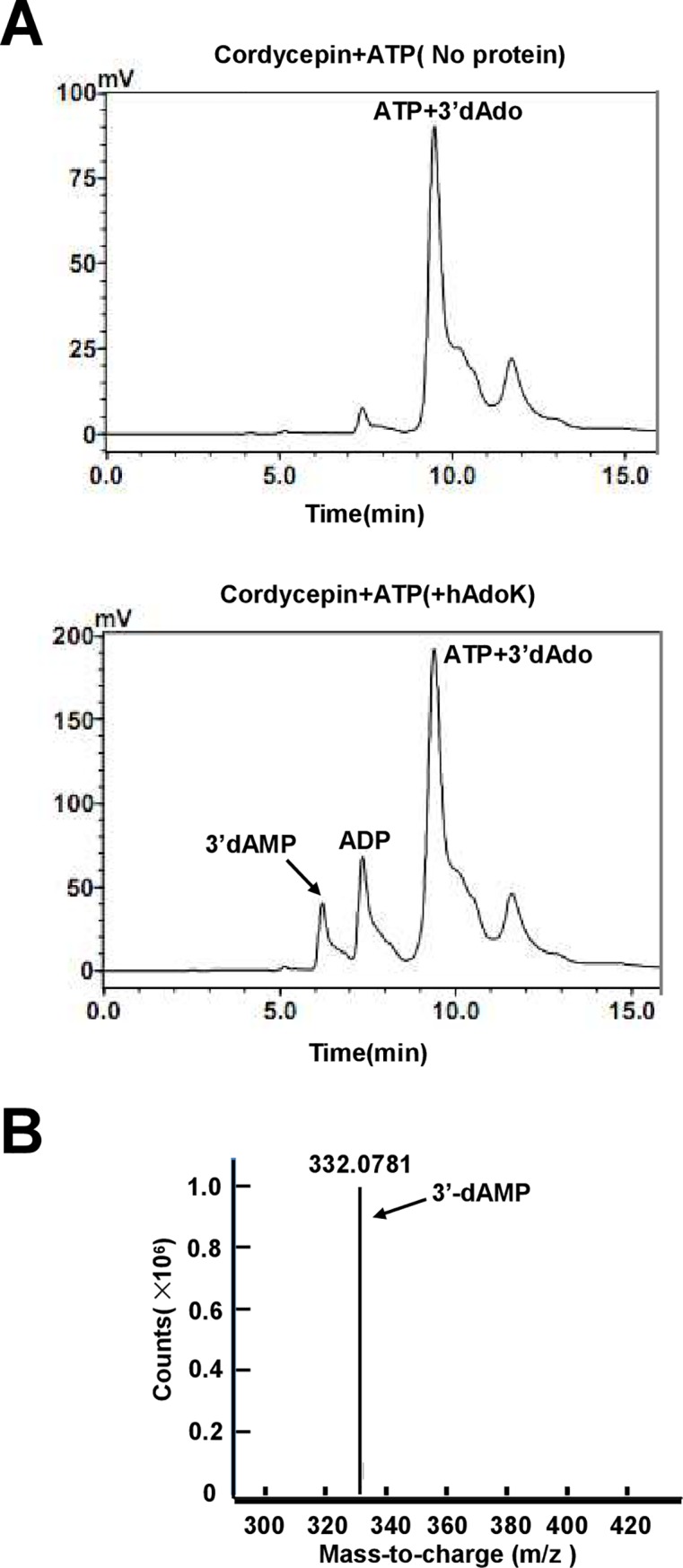
Assay for the new compound produced from cordycepin by human kinase. (A) HPLC separation of the new products from cordycepin. ATP and cordycepin were co-incubated with (lower panel) or without (upper panel) hAdoK. A new peak for the product on the left of ADP peak is indicated by an arrow. (B) LC-MS characterization of the new product. The mass spectra peak at m/z 332.0781 corresponds to 3′-deoxyadenosine monophosphate (3′-dAMP) in methanol and is indicated.

Further, we examined if cordycepin also similarly affected the kinase enzyme of human AdoK. In comparison, cordycepin did not inhibit the activity of hAdoK, whereas hAdoK can efficiently catalyze phosphorylation of cordycepin, indicating that cordycepin is a substrate of hAdoK (Fig 3B). We further isolated and identified the new phosphorylated products of cordycepin by hAdoK through HPLC and LC/MS assays. As shown in S3 Fig, However, when cordycepin was used as a substrate, no new product could be detected in the reactions under similar conditions (S3B Fig). This observation is consistent with above results shown in Fig 3. By contrast, when cordycepin was co-incubated with hAdoK and ATP, a new product nearing the ADP peak could be clearly observed (Fig 4A, lower panel). Using a liquid chromatography in combination with mass spectrometry (LC-/MS) assays, we further identified the new product as 3′-deoxyadenosine monophosphate (3′-dAMP) (Fig 4B).

Therefore, cordycepin does not inhibit hAdoK but acts as its substrate, and hAdoK can phosphorylate cordycepin to produce a new deoxyadenosine derivative, 3′-dAMP.

### Deoxycoformycin enhances the bactericidal activity of cordycepin in vitro

**Fig 5:**
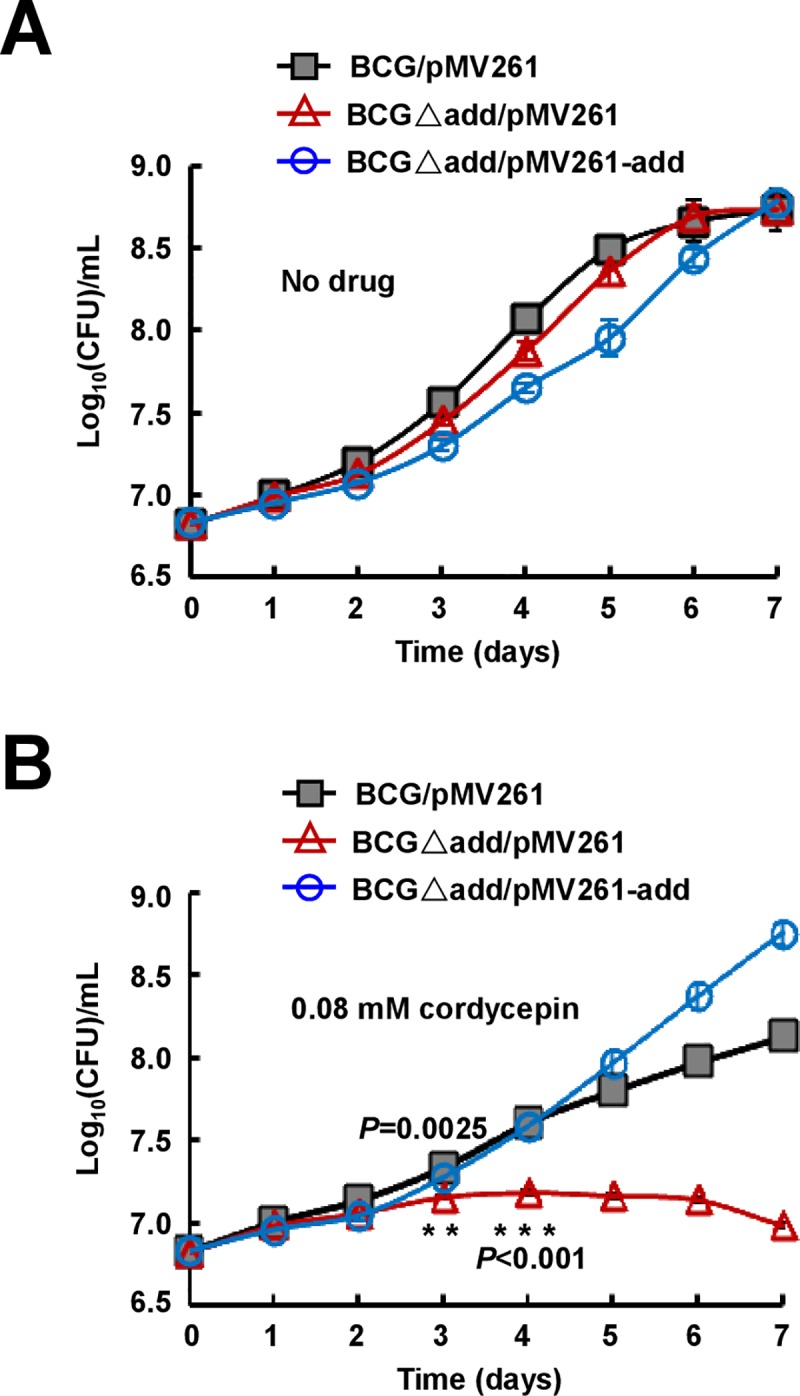
Effects of adenosine deaminase shunt on the mycobactericidal activity of cordycepin. Mycobacterial strains were grown in 7H9 medium without (A) or with 0.08 mM cordycepin (B). The *add*-deleted BCG strain is more sensitive to cordycepin than wildtype strain. Asterisks (*) denote a significant difference between the *add*-deleted and wildtype strain, and the *P*-values are indicated. The *P*-values were calculated by unpaired two-tailed Student’s *t*-test using GraphPad Prism 5.

**Fig 6:**
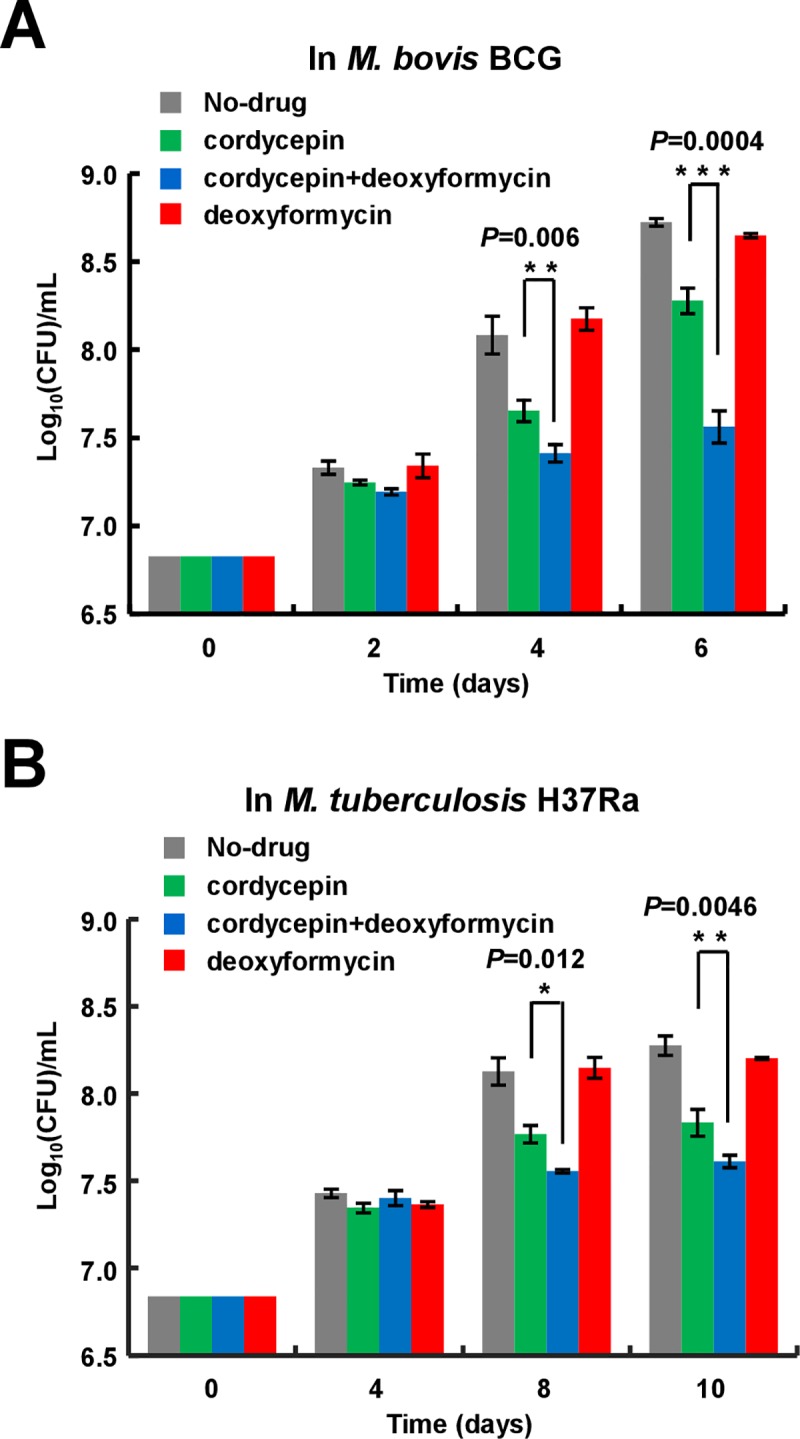
Effect of deoxycoformycin on the bactericidal role of cordycepin. *M*. *bovis* BCG (A) and *M*. *tuberculosis* H37Ra (B) strains were grown in 7H9 medium in the presence of either cordycepin or deoxycoformycin, or both drugs, and the CFUs were measured, respectively. All experiments were repeated three times. Error bars represent the SD in the figure. Asterisks indicate *P*-values (*, *P*<0.05; **, *P*<0.01; ***, *P*<0.001) from the unpaired two-tailed Student’s *t*-test using GraphPad Prism 5.

Cordycepin was previously shown to be hydrolyzed by adenosine deaminase (ADD) [36]. In *M*. *tuberculosis*, ADD is encoded by Rv3313c. To further dissect the bactericidal mechanism of cordycepin, we constructed *add*-deleted *M*. *bovis* BCG strain (S2A Fig) and its complemented strain (Δ*add*/pMV261-*add*), and then compared their differential cordycepin-sensitivities. As shown in Fig 5, the growth of *add*-deleted strain was more significantly inhibited by 0.08 mM cordycepin than that of wild type strain (Fig 5B, *P* = 0.0025 on day 3). This inhibition phenotype can be effectively rescued when the *add* gene was complemented into the *add*-deleted strain (Fig 5B). No significant growth difference was observed among the *add*-deleted (Δa*dd*/pMV261), complemented strain and the wild type strain in the absence of drugs (Fig 5A). These results implied that the deaminase could hydrolyze cordycepin and relieved its inhibitory effect on the mycobacterium. To further test this theory, we determined if an inhibitor of adenosine deaminase, deoxycoformycin, enhances the mycobactericidal effect of cordycepin. As shown in Fig 6, if compared to either cordycepin (0.06 mM) or deoxycoformycin (0.15mM) alone provided into the medium, co-use of these two drugs more effectively inhibited the growth of both *M*. *bovis* BCG (Fig 6A, *P* = 0.0004 on day 6) and *M*. *tuberculosis* H37Ra (Fig 6B, *P* = 0.0046 on day 10).

Taken together, our results suggest that deoxycoformycin, an inhibitor of adenosine deaminase, can enhance the bactericidal role of cordycepin.

### Deoxycoformycin enhances the bactericidal activity of cordycepin in host cell

**Fig 7:**
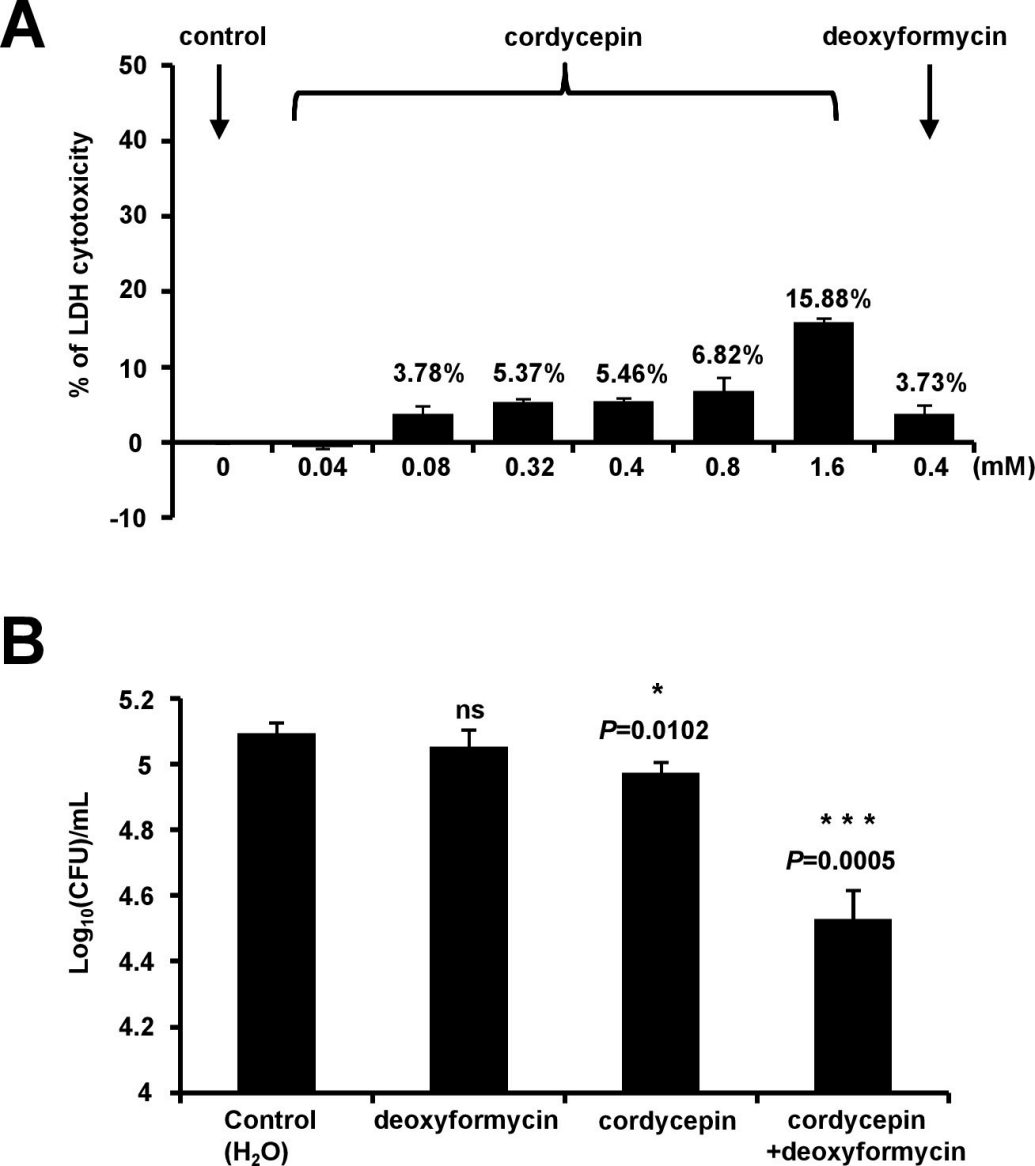
Effects of cordycepin and deoxyformycin on host cell and intracellular *Mycobacterium bovis* BCG. (A) Determination of LDH cytotoxicity of cordycepin or deoxyformycin in BMDMs. BMDMs (5×10^4^ cells per well) were plated in a 96-well plate and incubated overnight in an incubator at 37°C, 5% CO_2_. And on the next day, different concentrations of cordycepin or deoxyformycin were added to the culture media and incubated for 18 hours. LDH cytotoxicity was measured using the Pierce LDH Cytotoxicity Assay Kit. All experiments were repeated three times. Error bars are standard deviations. (B) BMDMs were infected with *Mycobacterium bovis* BCG (MOI = 1) and treated with 0.4 mM cordycepin or/and 0.4 mM deoxyformycin for 18 h, and the mycobacterial survival was assayed by determining colony-forming units (CFUs). All experiments were repeated three times. Error bars represent the SD in the figure. Asterisks indicate *P*-values (*, *P*<0.05; ***, *P*<0.001) from the unpaired two-tailed Student’s *t*-test using GraphPad Prism 5.

As cordycepin exhibited a dose-dependent mycobactericidal effect in vitro, and deoxyformycin can enhance the bactericidal role of cordycepin, we analysed the cytotoxicity of cordycepin and deoxyformycin to bone marrow-derived macrophages (BMDMs). As shown in Fig 7A, cordycepin shows no obvious cytotoxicity to BMDMs even the concentration was raised up to 0.8 mM (13×MIC of *M*. *bovis* BCG). And the deoxycoformycin also showed no obvious cytotoxicity to BMDMs when the concentration was 0.4 mM. Further, we determined bactericidal activity of cordycepin or/ and deoxycoformycin in host cell. As shown in Fig 7B, cordycepin (0.4 mM) can effectively inhibit the growth of intracellular *Mycobacterium bovis* BCG (*P*<0.05). By contrast, deoxycoformycin (0.4 mM) has no obvious effect on the growth of intracellular *Mycobacterium*. Interestingly, co-use of these two drugs more effectively inhibited the growth of the intracellular *Mycobacterium bovis* BCG (*P*<0.001).

Therefore, our results indicated that deoxycoformycin enhances the bactericidal activity of cordycepin in host cell.

## Discussion

Cordycepin is an efficient component of a traditional Chinese medicine, *Cordyceps spp*. As a nucleoside analogue, it has been confirmed to have a broad spectrum of biological activity [3–4, 6–8, 13, 37–38]. In the last few years, the anti-tumor activity and mechanism of cordycepin have been extensively studied [5, 10–12]. However, there is no document on its anti-bacterial mechanism to date. In the present study, we report that cordycepin has a good killing activity on slow-growth mycobacteria such as *M*. *tuberculosis* H37Ra and *M*. *bovis* BCG. An active AdoK kinase in mycobacteria is required for the bacterial sensitivity to cordycepin. We showed that cordycepin is an effective substrate of human AdoK kinase, whereas it competitively inhibited the activity of mycobacterial AdoK. Our study uncovered a potential killing mechanism of cordycepin on mycobacteria.

AdoK is a purine salvage enzyme that catalyzes the phosphorylation of adenosine to AMP [19]. The purine salvage pathway is one of the promising pathways for developing novel nucleoside analogs with anti-tuberculosis activity [18]. For example, 2-Methyladenosine (methyl-Ado) is a previously reported nucleoside analogue that selectively inhibits the growth of *M*. *tuberculosis* [22] and it is primarily converted to methyl-AMP by adenosine kinase *in vivo*. However, methyl-Ado is a better substrate for *M*. *tuberculosis* AdoK than for its homolog in human [19]. The anti-tuberculosis activity of the compound attributes to the differential substrate preference of their adenosine kinases in mycobacteria and human. In the present study, we found that cordycepin is a poor substrate of AdoK and it can competitively inhibit the catalytic activity of AdoK. Using a cordycepin-resistant mutant strain screening in combination with whole-genome sequencing method, we confirmed that either *adoK* -V33A or *adoK-*S115L mutation in *M*. *bovis* BCG resulted in loss of the mycobacterial sensitivity to cordycepin. Further, we found that both AdoK-V33A and AdoK-S115L mutant proteins almost lost their adenosine kinase activities (Fig 3A). And either *adoK*-S115L or *adoK*-V33A mutant gene could not affect the resistant phenotype of *adoK*-deleted *M*. *bovis* BCG to cordycepin. Therefore, the current study implied that an active AdoK kinase is required for the mycobacterial sensitivity to cordycepin, and AdoK is a potential target of cordycepin in *M*. *tuberculosis* and *M*. *bovis* BCG. Strikingly, an insoluble macromolecular structure could be clearly observed when a higher concentration of cordycepin was mixed with AdoK *in vitro* (S4 Fig), thereby preventing the use of a traditional Isothermal Titration Calorimetry assay to evaluate the physical interaction between cordycepin and AdoK. Although mycobacteria have additional purine nucleoside metabolism shunts such as adeenosine deaminase pathway [39, 40], it is proposed that mycobacteria preferentially use AdoK to directly produce AMP for further DNA or RNA synthesis [18]. Nevertheless, in the absence of *adoK* gene, mycobacteria can alternatively use adenosine deaminase or other pathway for survival. This is why *adoK*-deleted BCG is resistant to cordycepin. Our findings, together with previous observations, support a model, in which cordycepin selectively hijacks mycobacterial AdoK to form an uncharacterized lethal intermediate structure, which finally kills the mycobacteria. In this regard, an active AdoK kinase in mycobacteria is required for the mycobactericidal mechanism of cordycepin. By contrast, the human AdoK can efficiently catalyze phosphorylation of cordycepin to produce intoxic 3′-dAMP and, therefore, does not have a side effect.

Another interesting finding from the present study is that deoxycoformycin, an inhibitor of adenosine deaminase, can significantly enhance the bactericidal role of cordycepin. Adenosine deaminase widely exists in plants, mammals and bacteria, including *M*. *tuberculosis* [39, 41]. In mammalian cells, cordycepin was shown to be deaminated by their adenosine deaminases to form inactive metabolite 3′- deoxyinosine [41]. In the present study, we show that the *add*-deleted BCG strain is more sensitive to cordycepin than wild type strain (Fig 5), implying that cordycepin can also be hydrolyzed by ADD and partially lost its bactericidal activity in *M*. *bovis* BCG and *M*. *tuberculosis*. Consistently, an inhibitor of adenosine deaminase, deoxycoformycin, can significantly increase the mycobactericidal effect of cordycepin in vitro and in host cell (Figs 6 and 7). This finding supports well our model in which cordycepin kills mycobacterium by hijacking its AdoK, a major purine salvage enzyme utilized by *M*. *tuberculosis*.

**Fig 8:**
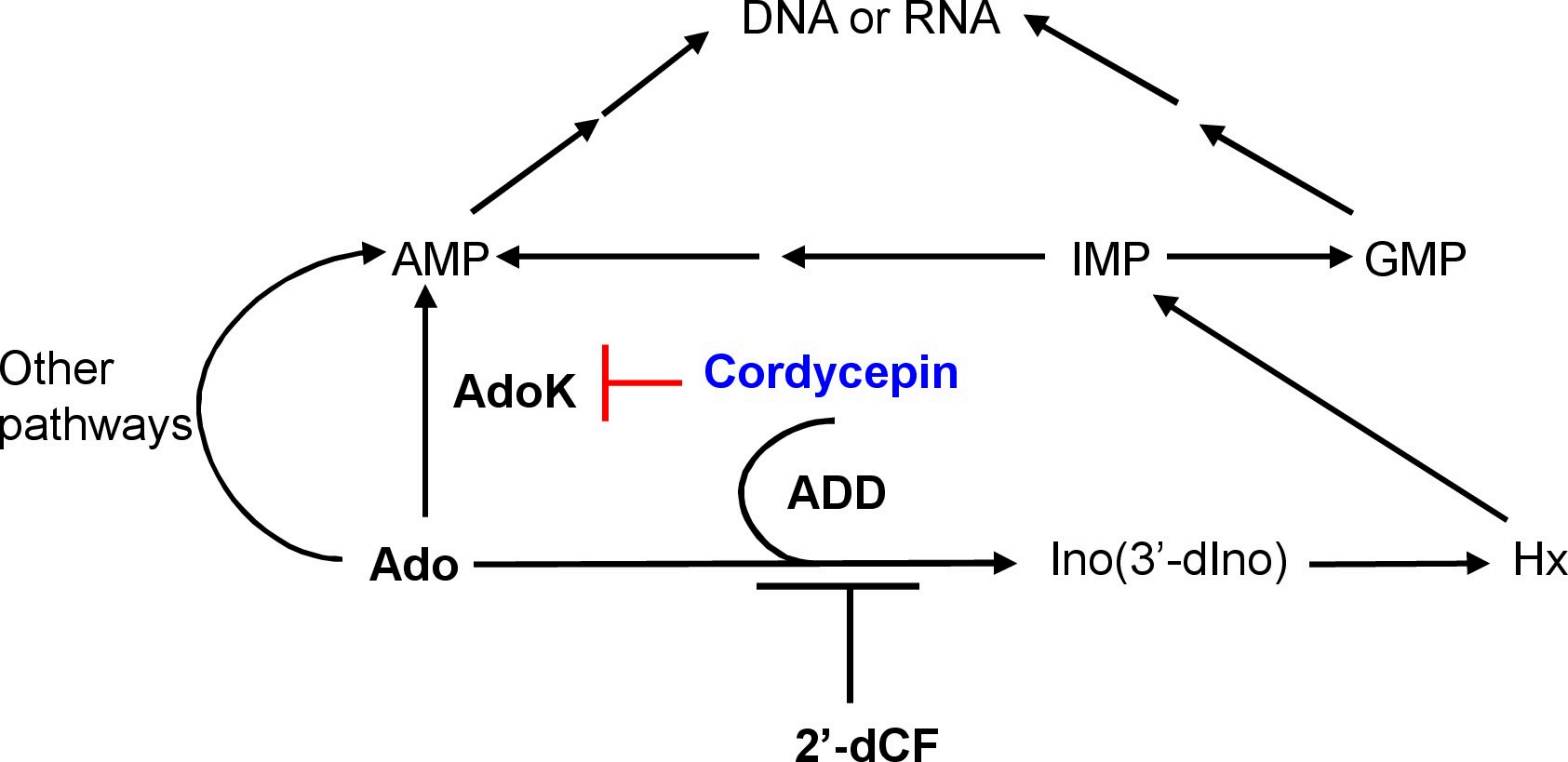
Metabolic pathways of Ado in *M*. *tuberculosis* and mycobactericidal mechanism of cordycepin. Ado is converted into AMP by adenosine kinase (AdoK) or inosine (Ino) by adenosine deaminase (ADD) in *M*. *tuberculosis*. Cordycepin targets AdoK to form a lethal intermediate structure. Deoxycoformycin (2′-dCF) inhibits the activity of ADD to enhance the mycobactericidal role of cordycepin.

Taken together, this study characterized cordycepin as a new mycobactericidal compound, and it can target at least two different enzymes in mycobacteria as shown in Fig 8, which results in totally different consequence. On the one hand, cordycepin can arrest adenosine kinase to produce potentially toxic intermediate product, which is lethal to *M*. *bovis* and *M*. *tuberculosis*. On the other hand, cordycepin can be hydrolyzed by adenosine deaminase to become inactive metabolite, and this pathway can be cut off by deoxycoformycin.

## Supporting information

S1 Fig Assays for the cordycepin-sensitivity of mutant CR02 and its *adoK* complemented BCG strain. Mutant CR02-V33Aselected on cordycepin was grown in 7H9 medium containing 0, 0.16, or 0.64 mM cordycepin at 37°C for 9 days. Samples were taken and the CFUs were measured. All experiments were repeated three times. Error bars are standard deviations.
(DOC)

S2 Fig Cordycepin sensitivity assays for the *S115L*-, *V33A*- complemented BCG strains. (A)PCR verification of *adoK* and *add* knockout strain PCR amplification was performed using the genome of knock out strain as a template. Lane 1, *adoK* (WT/BCG genome); Lane 2, *hyg* (WT/BCG genome); Lane 3, *adoK*(*adok*-deleted BCG genome); Lane 4, *hyg* (*adok*-deleted BCG genome); Lane 5, *add* (WT/BCG genome); Lane 6, *hyg*(WT/BCG genome); Lane 7, *add* (*add*-deleted BCG genome); Lane 8, hyg(*add*-deleted BCG genome). The lengths of genes were shown as follows: *adok*, 975bp; *hyg*, 999bp; *add*, 1098bp. (B) Assays for the sensitivities of two complemented strains to cordycepin. *S115L*-, *V33A*- complemented BCG strains were grown in 7H9 medium containing 0, 0.16, 0.32, and 0.64 mM cordycepin at 37°C for 9 days. Samples were taken and the CFUs were measured. All experiments were repeated three times. Error bars are standard deviations.
(DOC)

S3 Fig HPLC assays for the reaction products of Ado or cordycepin catalyzed by *M. tuberculosis* AdoK *in vitro*. Ado or cordycepin was used as a substrate and ATP as a phosphate donor Ado (A) or cordycepin (B) and ATP were co-incubated with (the lower panel) or without AdoK (the upper panel).
(DOC)

S4 Fig Microscopic observation for insoluble complex formation. 25 μM AdoK was mixed with 250 μM cordycepin and the visible white insoluble macromolecular can be observed. The macromolecular complex was assayed and magnified at 4×(overall panel) through Olympus optical microscope.
(DOC)

S1 Table Plasmids used in this work. (DOC)

S2 Table Summary statistics of whole genome sequencing. (DOC)
