# Supplementary material for: Cordycepin kills Mycobacterium tuberculosis through hijacking the bacterial adenosine kinase
Source: PLoS One. 2019 Jun 14;14(6):e0218449. doi: 10.1371/journal.pone.0218449 (PMC6568415; doi:10.1371/journal.pone.0218449)
Supplement: S1 Fig — Mutant CR02-V33Aselected on cordycepin was grown in 7H9 medium containing 0, 0.16, or 0.64 mM cordycepin at 37°C for 9 days. Samples were taken and the CFUs were measured. All experiments were repeated three times. Error bars are standard deviations. (DOC) [file pone.0218449.s001.doc]

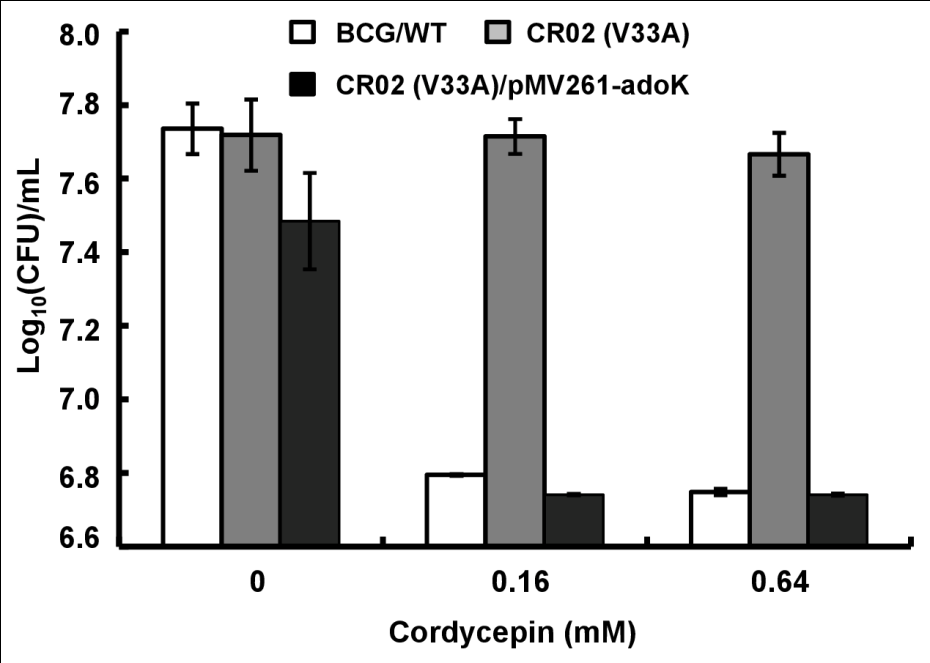


**S1 Fig. Assays for the cordycepin-sensitivity of mutant CR02 and its *adoK* complemented BCG strain.** Mutant CR02-V33Aselected on cordycepin was grown in 7H9 medium containing 0, 0.16, or 0.64 mM cordycepin at 37 °C for 9 days. Samples were taken and the CFUs were measured. All experiments were repeated three times. Error bars are standard deviations.
