# Supplementary material for: Cordycepin kills Mycobacterium tuberculosis through hijacking the bacterial adenosine kinase
Source: PLoS One. 2019 Jun 14;14(6):e0218449. doi: 10.1371/journal.pone.0218449 (PMC6568415; doi:10.1371/journal.pone.0218449)
Supplement: S2 Fig — (A)PCR verification of adoK and add knockout strain PCR amplification was performed using the genome of knock out strain as a template. Lane 1, adoK (WT/BCG genome); Lane 2, hyg (WT/BCG genome); Lane 3, adoK(adok-deleted BCG genome); Lane 4, hyg (adok-deleted BCG genome); Lane 5, add (WT/BCG genome); Lane 6, hyg(WT/BCG genome); Lane 7, add (add-deleted BCG genome); Lane 8, hyg(add-deleted BCG genome). The lengths of genes were shown as follows: adok, 975bp; hyg, 999bp; add, 1098bp. (B) Assays for the sensitivities of two complemented strains to cordycepin. S115L-, V33A- complemented BCG strains were grown in 7H9 medium containing 0, 0.16, 0.32, and 0.64 mM cordycepin at 37°C for 9 days. Samples were taken and the CFUs were measured. All experiments were repeated three times. Error bars are standard deviations. (DOC) [file pone.0218449.s002.doc]

**
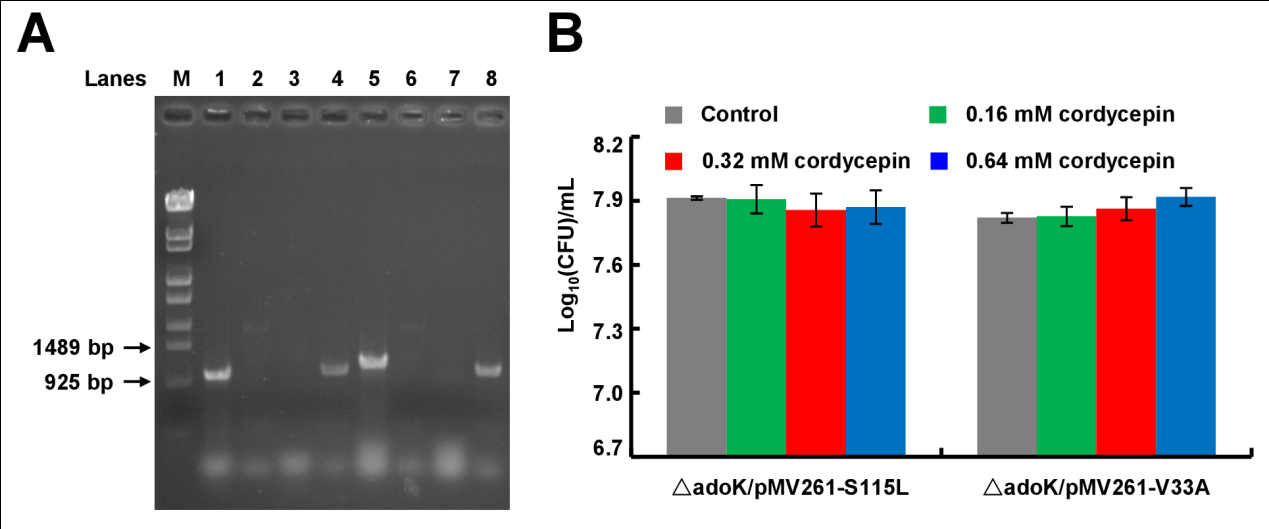
**

**S2 Fig. Cordycepin sensitivity assays for the *S115L*-, *V33A*- complemented BCG strains.** (A)PCR verification of *adoK* and *add* knockout strain PCR amplification was performed using the genome of knock out strain as a template. Lane 1, *adoK* (WT/BCG genome); Lane 2, *hyg* (WT/BCG genome); Lane 3, *adoK*(*adok*-deleted BCG genome); Lane 4, *hyg* (*adok*-deleted BCG genome); Lane 5, *add* (WT/BCG genome); Lane 6, *hyg*(WT/BCG genome); Lane 7, *add* (*add*-deleted BCG genome); Lane 8, hyg(*add*-deleted BCG genome). The lengths of genes were shown as follows: *adok*, 975bp; *hyg*, 999bp; *add*, 1098bp. (B) Assays for the sensitivities of two complemented strains to cordycepin*. S115L*-, *V33A*- complemented BCG strains were grown in 7H9 medium containing 0, 0.16, 0.32, and 0.64 mM cordycepin at 37 °C for 9 days. Samples were taken and the CFUs were measured. All experiments were repeated three times. Error bars are standard deviations.
