# Supplementary material for: Cordycepin kills Mycobacterium tuberculosis through hijacking the bacterial adenosine kinase
Source: PLoS One. 2019 Jun 14;14(6):e0218449. doi: 10.1371/journal.pone.0218449 (PMC6568415; doi:10.1371/journal.pone.0218449)
Supplement: S3 Fig — Ado or cordycepin was used as a substrate and ATP as a phosphate donor Ado (A) or cordycepin (B) and ATP were co-incubated with (the lower panel) or without AdoK (the upper panel). (DOC) [file pone.0218449.s003.doc]

**
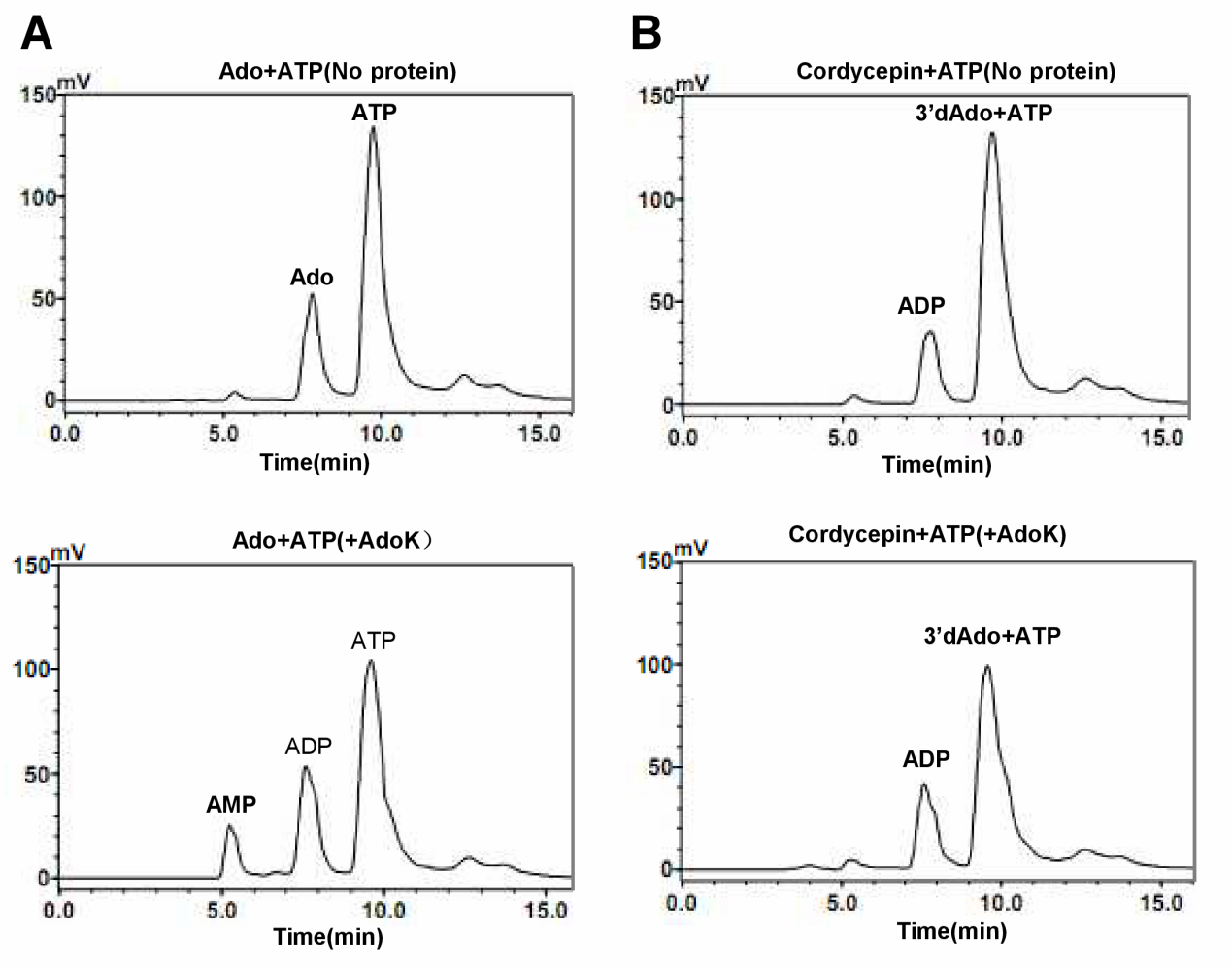
**

**S3 Fig.** **HPLC assays for the reaction products of Ado or cordycepin catalyzed by *M. tuberculosis* AdoK *in vitro*.** Ado or cordycepin was used as a substrate and ATP as a phosphate donor Ado (A) or cordycepin (B) and ATP were co-incubated with (the lower panel) or without AdoK (the upper panel).
