# Supplementary material for: Cordycepin kills Mycobacterium tuberculosis through hijacking the bacterial adenosine kinase
Source: PLoS One. 2019 Jun 14;14(6):e0218449. doi: 10.1371/journal.pone.0218449 (PMC6568415; doi:10.1371/journal.pone.0218449)
Supplement: S4 Fig — 25 μM AdoK was mixed with 250 μM cordycepin and the visible white insoluble macromolecular can be observed. The macromolecular complex was assayed and magnified at 4×(overall panel) through Olympus optical microscope. (DOC) [file pone.0218449.s004.doc]

**
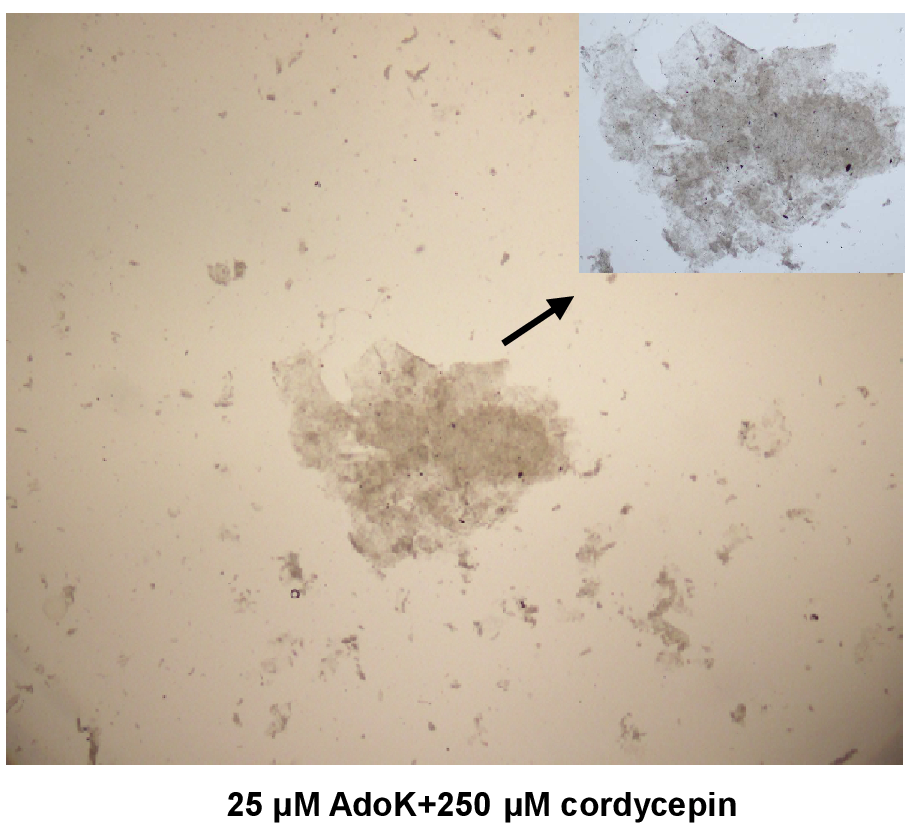
**

**S4 Fig. Microscopic observation for insoluble complex formation.** 25 μM AdoK was mixed with 250 μM cordycepin and the visible white insoluble macromolecular can be observed. The macromolecular complex was assayed and magnified at 4×(overall panel) through Olympus optical microscope.
