# Supplementary material for: Cordycepin kills Mycobacterium tuberculosis through hijacking the bacterial adenosine kinase
Source: PLoS One. 2019 Jun 14;14(6):e0218449. doi: 10.1371/journal.pone.0218449 (PMC6568415; doi:10.1371/journal.pone.0218449)
Supplement: S1 Table — (DOC) [file pone.0218449.s005.doc]

**S1 Table Plasmids used in this work.**

| Plasmid Relevant features Source | | |
| --- | --- | --- |
| pMV261-adoK | *adoK* gene cloned in pMV261 | This work |
| pMV261-adoK-S115L | *adoK* mutant S115L gene cloned in pMV261 | This work |
| pMV261-adoK-V33A | *adoK* mutant V33Agene cloned in pMV261 | This work |
| pMind-adoK | *adoK* gene upstream 1000 bp and downstream 946 bp cloned in pMind | This work |
| pET-adoK | *adoK* gene cloned in pET | This work |
| pET-adoK-S115L | *adoK* mutant S115L gene cloned in pET | This work |
| pET-adoK-V33A | *adoK* mutant V33Agene cloned in pET | This work |
| pET-hAdoK | Human *adoK* gene cloned in pET | This work |
| pMV261-MtbADD | Mtb-*add* gene cloned in pMV261 | This work |
| pMind-MtbADD | Mtb-*add* gene upstream 1000 bp and downstream 1000bp cloned in pMind | This work |
