# Supplementary material for: Cordycepin kills Mycobacterium tuberculosis through hijacking the bacterial adenosine kinase
Source: PLoS One. 2019 Jun 14;14(6):e0218449. doi: 10.1371/journal.pone.0218449 (PMC6568415; doi:10.1371/journal.pone.0218449)
Supplement: S2 Table — (DOC) [file pone.0218449.s006.doc]

**S2 Table Summary statistics of whole genome sequencing.**

| **Sample Name** | **Length** | **Covered Base** | **Total Base** | **Coverage (%)** | **Depth** |
| --- | --- | --- | --- | --- | --- |
| BCG | 4,374,522 | 4,374,518 | 3,596,282,062 | **100.00** | 822.10 |
| CR01 | 4,374,522 | 4,374,517 | 3,215,786,212 | **100.00** | 735.12 |
| CR02 | 4,374,522 | 4,373,641 | 2,460,370,076 | **99.98** | 562.43 |
